# A Holistic Approach for Ethics and Sustainability in the Food Chain: The Gateway to Oral and Systemic Health

**DOI:** 10.3390/foods13081224

**Published:** 2024-04-17

**Authors:** Theodoros Varzakas, Maria Antoniadou

**Affiliations:** 1 Department Food Science and Technology, University of the Peloponnese, 24100 Kalamata, Greece; 2 Department of Dentistry, School of Health Sciences, National and Kapodistrian University of Athens, 11527 Athens, Greece; 3 Certified Systemic Analyst Program in Systemic Management (CSAP), University of Piraeus, 18534 Piraeus, Greece

**Keywords:** food ecosystem, food chain, food morality, ethics, oral health, systemic health, diet habits, spirituality, sustainability

## Abstract

Food production is a complex matter, affecting people’s lives, organizations’ profits, and the well-being of the whole planet, and has multifaceted ethical considerations surrounding its production, distribution, and consumption. This paper addresses the pressing need to confront ethical challenges within the food system, encompassing issues such as environmental sustainability, food security, and individual food choices for better oral and systemic health of all individuals around the globe. From agricultural practices to global trade and food waste, ethical implications are addressed across various domains, highlighting the interconnectedness of ethical decision-making in the food industry. Central themes explored include the ethical dimensions of food production methods, the impact of global trade on food ethics, and the role of individuals in making ethically informed food choices. Additionally, this paper considers the spiritual and physical significance of food, particularly through the lens of oral health as a gateway to holistic well-being. Recognizing the complexity of the food and mouth ecosystem, this paper calls for serious interventions in legislation and economics to promote ethical protocols and techniques for sustainability reasons. It emphasizes the importance of ethical considerations in food safety management systems, regulatory frameworks, and quality standards. Moreover, this paper underlines the need for a comprehensive approach to address ethical dilemmas and moral values inherent in the food industry and oral health policies, adopting the precautionary principle and ethical decision-making frameworks. This article finally aims to serve as a call to action for stakeholders across the food industry and the healthcare sector, to prioritize ethical practices, promote transparency, rearrange economic parameters, and work towards a more sustainable and equitable food system for inner and outer oral and systemic health and human sustainability for all.

## 1. Introduction

In contemporary society, the food ecosystem stands as a complex system involving environmental, social, economic, and ethical dimensions [1]. The production, distribution, and consumption of food are not merely utilitarian processes but rather intricate webs of interconnectedness that profoundly impact individuals, communities, and the planet at large [2]. At the heart of this multifaceted system lies ethical considerations that permeate every aspect of the food chain [3].

Human attitudes towards animals have been influenced originally by the ancient Greek philosophies addressing the formulation of terms such as ethos (ἦθος, ἔθος), ethics (δέον), and moral (ευδαιμονία). “Ethos” is defined as the character, sentiment, or disposition of a community or people, considered as a natural endowment. “Ethos” is a Greek word corresponding roughly to “ethics” [4]. Food ethics is not a new field, it always existed, but it appears today as a high priority, moving beyond the traditional form of ethics, as described by Monterrosa et al. [5]. Under the definition of morality [6], food morality attempts to bridge the gap between our ethical values and our food-related behaviors. This means that there are moral implications in our everyday food choices [3]. Moral considerations accompanying the production, distribution, and consumption of food can be considered as food morality [7]. The application of ethical principles in conjunction with the ethical production/manufacturing of food along with the environmental and social impacts on our diets, animal welfare, farmers, and the whole society is food morality, according to Hernandez et al. [8]. The concept of food morality encompasses issues such as climate change, resource depletion, social inequalities, and public health. A holistic approach has been considered in food research [9] on the reduction in food loss and waste [10] and on food type interdependence [11], food intake, and appetite [12]. The transformation of food environments nowadays, along with the change in the dynamics of lifestyles, implies a theory of holistic food ethics. Food ethics considers mainly animal ethics and positive and negative views regarding animal rights [13] and animal suffering [14], taking also into account environmental ethics, which analyses the sustainability of food systems [15]. Furthermore, social justice examines the nutritional side of foods along with access to them, according to Ruben et al. [16]. The adoption of vegetarian or vegan diets, consumption of organic food, or avoiding buying foods from companies adopting controversial practices means alignment of moral values with food choices [11,17]. Most people agree on the non-moral neutrality of food choices nowadays. Indeed, the relevance of food morality is greatly depicted by the heavy industrialization of food systems with negative social and environmental impacts including pollution, biodiversity loss, climate change, and labor exploitation. Model sustainability and equitability [18] are in great need today in conjunction with morality.

This study aims to provide a comprehensive examination of the ethical dimensions inherent in the modern food system, with a specific focus on the pivotal role of oral health and its interconnectedness with broader ethical considerations. Through a multidisciplinary lens encompassing ethics from Socrates to Spinoza, environmental science, oral and general health sciences, sociology, anthropology, and spirituality, our objective is to explore the area of ethical dilemmas that permeate every aspect of the food ecosystem from production to the mouth, the gateway to the whole body. From analyzing agricultural practices and production methods to investigating global trade dynamics, health economics, regulatory frameworks, and quality tools in the ethical management of the food ecosystem, we seek to uncover the moral complexities and challenges faced at each stage of the food chain. Furthermore, we aim to shed light on the often-overlooked influence of individual food choices on the ethical base of our food system, recognizing their profound impact on sustainability, social justice, and public health. We then discuss the ethical imperatives of food safety, quality standards, and the pervasive issue of food fraud. We finally examine the significance of oral health as the gateway to systemic health and the mouth as the gateway to the human body to stimulate meaningful dialogue and inspire collective action toward building a more ethical and sustainable food system that nurtures the holistic well-being of individuals and communities alike. 

## 2. Methodology of this Review

Given the multifaceted nature of the theme and the vast body of literature available, our approach was designed to systematically sift through the variety of information to offer a comprehensive understanding of the ethical complexities surrounding food production, distribution, consumption, and sustainability. Firstly, we clearly outlined the specific ethical challenges that were about to be addressed, focusing on the multifaceted nature of the food ecosystem. These challenges included issues related to (1) morality vs. ethics, (2) cultural considerations in food ethics, (3) ethics in religious food, (4) food and spirituality issues, (5) food dynamics, (6) GMO ethics and neophobia, (7) food regulation, (8) food safety and quality standards, (9) food fraud and ethical production practices, (10) consumer rights and behavior, (11) the mouth and ethical food, (12) significance of oral health in the food chain, (13) economic implications and ethics in the prevention and/or provision of oral health, and (14) sustainability issues in the food chain up to the mouth gateway.

Then, in this review study, we employed a methodological approach that encompasses several key steps aligned with the theme of the ethical dimensions inherent in various facets of the food system. Firstly, we selected databases that cover a wide range of disciplines relevant to the topic, including PubMed, Web of Science, Scopus, and Google Scholar. Next, we developed a comprehensive search strategy using relevant keywords and phrases related to food ethics, sustainability, morality, and related concepts. We considered using a combination of controlled vocabulary (e.g., MeSH terms in PubMed). In this sense, for “sustainability of systems through food”, we used the following terms: food supply/supply and distribution, food industry/economics, food preservation/methods, food handling/methods, food technology/methods, food packaging/methods, sustainable agriculture/methods, agriculture/economics, environmental sustainability, and conservation of natural resources. For “health economics“, we used the following terms: “health economics”, “health expenditures”, “healthcare costs”, “cost-benefit analysis”, “cost control”, “economic models”, “health services accessibility”, “health policy”, “health insurance”, and “managed care programs”. Also, for “oral health” issues, we used the following terms: “oral health”, “dental health services”, “dental health surveys”, “periodontal diseases”, “tooth diseases”, “dental caries”, “oral health promotion”, and “dental public health”. Finally, we searched additional free-text terms such as “food ethics”, “ethical production”, “food sustainability”, “food morality”, “ethical consumption”, “food waste ethics”, “animal welfare”, environmental ethics”, “oral health”, “general health”, and “diet habits”. In the end, we linked ethics with food consumption behavior.

We also defined clear inclusion/exclusion criteria based on publication date range, language, and study design, ensuring that the selected literature aligns with the objectives of this review, and extending the search from January 2000 to 31 March 2024. Following this, we conducted an initial screening of the search results based on titles and abstracts to identify potentially relevant articles, followed by a detailed review of full-text copies to assess the relevance and quality of those selected in the first place. We considered certain cases including grey literature sources such as reports and policy documents considering the nature of the theme. Additionally, we conducted manual searches of relevant journals and the reference lists of the included articles to identify additional sources. Upon data extraction, we synthesized the findings to identify common themes, patterns, and gaps in the literature. Furthermore, a quality assessment of the final included studies was conducted, and a narrative synthesis of the literature was prepared, highlighting key findings and implications. 

## 3. Morality versus Ethics in the Food Industry

Morality is defined as the actions of people in their relationships with others [19]. This means responsibility for their actions and their engagement with others, making all co-responsible for social construction, according to Dasuky [20]. The latter author believes that ethics and morality are closely interrelated, somewhere between the passive and the dynamic or between living just for living and the longing to live well [20].

In contrast to Huxley [21], who reported on the selfishness and amorality of human nature, Hauser [22] argues about the human innate moral faculty shared by other primates, guiding and separating moral judgments from nonconscious forms. Hauser discusses a moral organ allowing for the acquirement of the moral system we wish to develop. This is in conjunction with de Waal and Aureli [23], illustrating the judgmental ability of chimpanzees regarding the consequences of certain actions in great resemblance to true moral judgments. Hauser [22] also postulates that our moral judgments are determined by a biological “moral grammar”. The prohibition of murder or the promotion of reciprocity constitute basic moral principles that are shared by all cultures. On this same point, Socrates, the ancient Greek philosopher, states that “No one does evil willingly”, but this notion is a misunderstanding stemming from loose translations and misinterpretations. In Plato’s work, “Protagoras” (358d) [24]), Socrates articulates a very different position. What he emphasizes is that no person commits evil willingly, nor do they choose to pursue what they believe to be evil. In this text, Socrates examines the nature of evil and good, concluding that the perception of evil is not real, as evil does not exist as an independent force in the universe. Through the distinction between evil and good, one can achieve a virtuous state. 

The exploration of ethics, as discussed in the context of Socratic philosophy, can be extended to the field of food ethics. Just as Socrates examines the nature of good and evil and the conscious choice behind actions, food ethics involves considering the moral implications of food production, consumption, and distribution. In the same way that Socrates suggests that people do not knowingly choose evil [24], proponents of food ethics argue that individuals may not always be fully aware of the ethical consequences of their food choices. For example, someone might unknowingly support unethical agricultural practices or contribute to environmental degradation through dietary habits. So, by applying the Socratic principles of moral inquiry and self-awareness, individuals can engage in more conscientious decision-making regarding food. This might involve considering the environmental impact of food production, the treatment of animals in agriculture, or the social justice issues related to equal food access and distribution. In essence, just as Socrates encourages individuals to critically examine their actions and motivations [24], food ethics should encourage a similar introspection regarding the ethical dimensions of food choices and consumption patterns.

The interconnectedness between human welfare, respect for animal life, and the environment affecting our food choices is well recognized by different philosophical theories such as utilitarianism, Kantianism, natural rights, and virtue theory [3]. Ultimately, everyday choices that affect our lives and the world we share have to do with food morality and ethics. The nourishment of our bodies but also our souls with choices reflecting our deepest values and contribution to building a more just and equitable world comes from an ethical approach to food. Deeper values of justice, respect, and moral excellence might arise from an ethical reflection on food. 

More specifically, utilitarian thinking refers to the Chinese philosopher Mozi, who lived between 490 and 403 BC. His work was later developed by Western thinkers such as Jeremy Bentham (1748–1832), John Stuart Mill (1806–1873), and Henry Sidgwick (1838–1900) [25] and has profound implications for the food industry’s approach to safety and ethics. According to utilitarianism, the contribution of actions to the general good or overall happiness corresponds to ethical value [25]. Within the context of food morality, this perspective emphasizes the importance of assessing the utility or happiness derived from food choices and consumption patterns. The concept of “utility” or the “greatest happiness principle” serves as the foundation of moral evaluation, as actions are deemed right insofar as they contribute to the promotion of happiness, as reported by West [26]. This implies that food production, distribution, and consumption practices should prioritize the well-being and satisfaction of individuals and society. From a safety standpoint, utilitarian thinking calls for ensuring that food products are safe for consumption and free from harmful contaminants or hazards. This aligns with the notion that the promotion of happiness and well-being requires safeguarding public health and minimizing risks associated with foodborne illnesses or adulteration. Furthermore, ethical considerations in the food industry are intertwined with utilitarian principles, as decisions regarding sourcing, production methods, and marketing should aim to maximize overall happiness and minimize harm. This includes prioritizing sustainable and ethical sourcing practices, promoting fair labor conditions in the agriculture and food industries, and minimizing environmental impact throughout the supply chain. If the food industry adheres to utilitarian principles, it can strive to maximize the overall well-being and happiness of individuals while also upholding ethical standards and ensuring the safety of food products [25,26].

In addition, Kantian ethics, rooted in the philosophical reflections of Immanuel Kant, emphasizes the centrality of rationality in determining moral principles and duties [27]. Kantism asserts that ethics are grounded in the inherent rationality of human beings and the notion of duty [27]. Granja [28] posits that moral convictions necessitate freedom of choice to be valid, underscoring the importance of autonomy in ethical decision-making. Within the area of food ethics, Kantian principles find application in addressing the fair treatment of animals and upholding ethical standards in the food industry. Moreover, the rationality inherent in human nature serves as a guiding force in determining our actions, leading to opposition to practices such as animal abuse and exploitation. Kantian ethics impel individuals to recognize the intrinsic worth and dignity of sentient beings, promoting the adoption of ethical animal rights and welfare practices in food production and consumption. Aligning with Kantian principles means that individuals and industries within the food sector are compelled to uphold their moral duty to treat animals with respect and compassion, thereby guaranteeing a more ethical and human approach to food production and consumption [29]. Moreover, Kantian ethics prompt individuals to reflect on the consequences of their food choices and to consider the broader implications of their actions on both human and non-human stakeholders in the food system. Thus, Kantian principles could serve as a framework for guiding ethical behavior and decision-making in the food industry and shaping conscientious food habits that prioritize respect for all living beings.

The natural rights theory also parallels the fact that natural rights derive from the creation of certain natural laws by God, or the idea that human nature is the origin of natural laws. Therefore, human rights are considered inherent to human nature [30]. Food choice, as a decision made by every individual, involves the exercise of willpower, which is considered our divine nature, as reported by Atteshli-Theotoki [31]. This innate capacity can empower us to resist anything toxic or harmful to our health, as well as counteract any negative tendencies. It is not a matter of fighting our weaknesses but understanding them and convincing ourselves and our subconscious that we can master them and disregard them. In this direction, each person has the choice to select a diet based on the value of individual freedom and autonomy [32], and this includes the consumption of meat. Finally, individual well-being derives from freedom of choice and self-determination, which are associated with personal development and making informed decisions about one’s diet [33].

Additionally, according to Cruz [34], the idea of natural rights links these rights to either divine decree or inherent human nature. This suggests that these rights are not given by human institutions. Kelsen [35] further elaborates on this concept by attributing the source of natural law to the essential nature of humanity, which serves as the ultimate authority in establishing rules and norms. Within this framework, the maintenance of a peaceful/mutual relationship with nature and all living beings assumes paramount importance, as reported by Lugo-Morin et al. [36,37]. This perspective underscores the recognition that the world and all manifestations of life are imbued with inherent value and are created by divine entities, reflecting the absolute wisdom, power, and love inherent in the universe [31]. Humanity must acknowledge and uphold the profound interconnectedness of the cosmos and all manifestations of life, which are intricately crafted by the Holy Archangels, reflecting boundless wisdom, power, and love [31]). Governed by the Circles of Possibilities inherent in each living entity, creation unfolds with divine intentionality and consciousness. Recognizing our role as co-creators, we must conscientiously control our thoughts, desires, and actions through introspection, analytical inquiry, and a commitment to truth [31]. As stewards entrusted with the care of creation, humans are further tasked with aligning their conduct with the principles of introspection, truth-seeking, and reverence for the intrinsic worth of all life forms. In the field of food ethics, this perspective underlines the imperative to honor the sanctity of nature and the inherent rights of every living being. Embracing these principles may guide our choices and behaviors towards empowering a more sustainable and ethical relationship with the natural world, thereby fulfilling our responsibility as stewards of the earth [31].

Furthermore, virtue theory lies in the development and inclusion of personal virtues and moral excellence in all aspects of human life [38]. In ancient Greece, Plato and his pupil Aristotle (384–322 BC) argued that morality has to do with leading a good life and being a morally good person [38]. Aristotle argued further that to become a good man (or a good woman), you must do what a good man does, according to Berti [39]. Aristotelian virtue ethics is a human-centered theory that relies on people and their characters and morality has more to do with the question “How ought I to be?” and does not deal with the morality of actions [40]. Virtue ethics reports that the power of striving to achieve the goal of virtue is morality and belongs to a teleological theory. However, a question arises regarding addressing ethical dilemmas where virtues compete following a viable decision-making procedure [30]. This concept of virtue ethics provides a lens through which to explore the complexities of food morality. Following this option, virtue ethics posits that morality entails the cultivation of virtuous character traits and the pursuit of virtuous goals [30]. This framework underscores the importance of striving towards ideals such as human well-being, respect for animal life, ecological sustainability, and social justice within the context of food morality. However, virtue ethics also acknowledges the inherent challenge of exploring ethical dilemmas where competing virtues pull in different directions [41]. In the field of food morality, this may manifest in situations where the pursuit of one ethical principle, such as environmental sustainability, conflicts with another, such as social equity in food access. While virtue ethics provides a robust foundation for guiding moral conduct, its lack of a concrete decision-making procedure poses a significant challenge in resolving such dilemmas [42]. Thus, applying virtue ethics to food morality necessitates careful consideration of how to balance competing virtues and reconcile conflicting ethical imperatives in the complex landscape of the food system.

Additionally, Lugo-Morin [3] urges us to integrate diverse considerations that encompass the lives of human beings, their respect for animal life, fundamental rights, environment, and social justice in creating a moral framework for food that goes beyond just eating for survival. Such a holistic approach challenges us to move beyond rigid dogmas and simplistic solutions, emphasizing the importance of fostering ties of solidarity among all who gather around the table. Indeed, the act of eating extends beyond the physical nourishment of bodies; it catalyzes nurturing relationships—most notably, our connection with the divine, whether conceived as God, the Creator, or the spiritual foundation that underpins everyone’s existence. This viewpoint emphasizes the deep importance of food beyond just providing nourishment, emphasizing its ability to strengthen spiritual connections and foster community bonds, thus enriching the human journey [3].

Ethical values dominate people’s lives [43,44,45]. Values can be defined as the sum of the positive/good properties that reflect the importance of a good person or thing, and these properties should be acquired and recommended [46,47]. There are different forms of values based on (1) material and economic issues, (2) politics, (3) social welfare (e.g., love, friendship, cooperation, and peace), (4) aesthetic conditions, (5) natural aspects of life (e.g., life, health, and nature), and (6) moral/ethical values (such as responsibility, honesty, conscientiousness, self-awareness, self-control, and dignity) [48]. The hierarchy of these values largely depends on the cultural environment [49] and may be understood differently both in terms of concepts of different values and significance [50,51]. Values in the food chain are not merely abstract concepts but are deeply embedded in the fabric of everyday life, influencing the actions and decisions of all stakeholders involved. Ethical values, as elucidated by Harris [52] and Kanungo [53], play a fundamental role in shaping the behavior and practices within the food industry. Within this framework, there exists a hierarchical structure of values, guided by rational or explicit rules set forth by the group, as noted by Landau and Osmo [54]. This hierarchy reflects the varying degrees of importance attributed to specific values within the broader system–society–culture complex, as highlighted by Jackson [50]. Particularly significant are the values that uphold the principles of human life, freedom, and justice, irrespective of contextual frames, as emphasized by Donnelly [55] and Schwartz and Bardi [56]. In the food chain, these values manifest in various forms, influencing decisions related to production methods, distribution practices, and consumer choices, thereby shaping the ethical contours of the entire ecosystem [55,56]. 

Finally, Spinoza’s “ethics” provides us with profound insights into human nature, ethics, and the pursuit of a good life, as discussed by Curley [57]. While Spinoza’s work may not directly address the food industry or diet and oral health initiatives, his philosophical principles can be applied to these areas to promote sustainability. Firstly, Spinoza emphasizes the interconnectedness of all things in the universe, thus providing us with the needed spiritual base. Applied to the food industry, this principle highlights the importance of recognizing the interdependence between food production, consumption, and environmental sustainability, as analyzed by Costa Deprá [58]. It may encourage stakeholders in the food industry to consider the broader ecological impact of their actions, promoting sustainable practices that minimize harm to the environment. Secondly, Spinoza’s ethics underscore the importance of caring for oneself and others. In the context of diet habits and oral health initiatives, this translates into promoting preventive care and education to empower individuals to take responsibility for their oral health and diet, promoting well-being and sustainability, as reported by Vikram et al. [59]. Then, Spinoza posits that humans are driven by the desire to increase joy and decrease suffering. Applied to the food industry, this principle calls for practices that prioritize the well-being of all stakeholders, including consumers, producers, and the environment, according to Viles et al. [60]. Sustainable food production methods that prioritize animal welfare, reduce food waste, and promote equitable access to nutritious food contribute to maximizing joy and minimizing suffering. He also emphasizes the role of reason in guiding human behavior. In the food industry, rational decision-making involves considering the long-term consequences of production methods, supply chain practices, and consumption patterns [61]. So, by employing critical thinking and evidence-based approaches, stakeholders can make informed decisions that promote sustainability and ethical food systems. His philosophy also celebrates diversity and encourages inclusivity. Overall, applied to the food industry, this principle calls for respecting cultural food traditions, promoting diverse agricultural practices, and ensuring equitable access to nutritious food for all communities. Embracing diversity and fostering inclusive food systems is the key for stakeholders to promote sustainability and social justice [29]. 

## 4. Cultural Considerations in Food Ethics

Cultural considerations play a pivotal role in shaping food ethics, with perceptions of morally acceptable food varying significantly across different cultures, as shown by Mardian et al. [62]. What may be seen as traditional and culturally appropriate food practices in one society, such as different types of meat consumption or the eating of insects, may be met with skepticism or rejection in another [63]. These variations reflect the influence of culture and tradition on dietary norms and behaviors within each community, as discussed by Murcott [64]. Furthermore, cultural and religious diversity underlines the importance of respecting the culinary practices and food traditions of various communities, affirming individuals’ rights to make decisions aligned with their cultural and religious beliefs [33].

Moreover, food aid derived from international and culturally different parts of humanity serves as a vital component of humanitarian assistance for individuals facing dire circumstances, such as immigrants and people living in war-torn regions (Tranchant et al., 2019) [65]. Designed to bolster food security and contribute to civil peace in destination countries, food aid plays a crucial role in addressing immediate needs and embeds ethical dilemmas. For example, studies examining the relationship between food aid and conflict present different conclusions [66,67]. While some research suggests that food aid may prolong armed conflicts, others argue that it reduces both the incidence and duration of conflicts [68,69]. The complexity of this relationship underscores the need for further analysis, with scholars identifying shortcomings in existing studies that raise questions about the validity of their findings [68,70,71,72]. Despite these challenges, it is vital to recognize that food aid serves as a lifeline in humanitarian endeavors, yet its impact hinges on meticulous assessment and continuous trial to uphold ethical standards and avert unforeseen repercussions.

## 5. Ethics in Religious Foods

Gillian Feeley-Harnik’s seminal work, “Religion and Food: An Anthropological Perspective” by Feeley-Harnik [73], has profoundly influenced scholarly discourse on the intersection of religion and food. Feeley-Harnik’s exploration emphasized the dynamic and transformative nature of food, challenging the notion of food as a static or natural symbol [73]. This perspective relates to the profound significance of food, eating, and fasting in shaping the beliefs and practices of diverse religious communities [73]. However, Feeley-Harnik’s analysis also highlighted a notable gap in scholarly inquiry, as religion itself often remained unexamined and implicitly understood [74]. Building on Feeley-Harnik’s insights, recent studies have begun to explore the multifaceted relationship between food and religion, offering serious perspectives on the role of food in shaping religious beliefs and practices [75,76]. According to these insights, religious food practices offer a profound glimpse into the moral, cultural, and ethical fabric of societies, often intersecting with legal and constitutional frameworks, as discussed by Pomeranz and Brownell [77]. For instance, the prohibition of certain foods, such as pork, may stem from moral or religious beliefs, necessitating careful consideration of public health, food safety, and ethical concerns, as shown by Lopez-Garcia [78]. This complex issue involves inspecting a delicate balance between upholding individual rights, cultural and religious freedoms, and the imperative to protect public health. It is important to mention that measures aimed at safeguarding public health are underpinned by robust scientific evidence regarding potential risks [78]. Moreover, in pluralistic societies, respect for cultural and religious diversity should be a guiding principle, ensuring that legal and regulatory measures are sensitive to the beliefs and practices of different communities [33]. Thus, religious freedom and conscience in many countries should be seriously considered before banning food for religious reasons [33]. 

Furthermore, religious dietary practices often entail periods of fasting or restriction of certain foods, deeply intertwined with spiritual and cultural traditions [79,80]. For example, the Greek Orthodox Church prescribes fasting during significant periods preceding Christmas, Easter Lent, and the Assumption, with specific guidelines on food consumption during these periods [79,80]. Similarly, adherence to Kosher and Halal dietary laws aligns with religious prescriptions, reflecting the intersection of faith and food [79,80]. There are also multiple possible versions of food having sacred significance in other civilizations. For example, Meigs [81] explained the rules regarding food among the Hua of Papua, New Guinea. The central notion has to do with nu, essence, which is transferred when touching and preparing food. Thus, the Hua have an elaborate set of rules about who can prepare food for whom, and who can eat what foods. An example is that mature, initiated males cannot eat leafy green vegetables that were picked by their real or classificatory wives.

Buddhist traditions, on the other hand, advocate for abstention from killing and the consumption of animal products, promoting a vegetarian way of eating as a means of fostering balance and spiritual well-being [82,83,84]. This dietary philosophy emphasizes principles of compassion, purity, and connection with the divine, underscoring the profound spiritual significance attributed to food practices, as described by Testoni et al. [85]. Similarly, within Christian traditions, the belief in humans being created in God’s image underscores the importance of seeking peace and harmony with all creatures, shaping dietary choices and ethical considerations accordingly, as noted by Suzworsky [86]. Some report that this sort of behavior promotes prayer, deification, purity, and contact with the divine [85]. Hence, the assumptions that humans were created in God’s image predicate Christian ideas, and some claim that peace should be sought among all creatures, especially when food preparation and consumption are involved [86].

## 6. Fasting and Vegetarians/Vegans

Another important argument of Christians who follow a strict vegetarian diet is fasting, which is God’s first and oldest commandment. Jesus further emphasized its value when He said: “This generation shall not be brought forth except in prayer and fasting”, meaning that “by fasting we fight our passions and make our human nature sensitive to His love and grace”. In the religion of Christianity, and especially the Orthodox religion, about 50% of the calendar year (180–200 days) pushes believers to abstain from meat, dairy, and eggs, as reported by McPherson [87].

“Veganism”, or “strict vegetarianism”, is a philosophy and lifestyle that aims to avoid the use of animals for food, clothing, or any other purpose and is based on a diet of plant foods. It entails a commitment to abstain from eating products of animal origin, including meat, fish, seafood, eggs, dairy products, and honey. The majority of followers of this lifestyle extend its practice to other aspects such as clothing, toiletries (e.g., not using beeswax, lanolin, or creams with animal fats), and cosmetics (not animal-based and those not tested on animals) [88]. Overall, the spirit behind veganism is rooted in ethical concerns, environmental awareness, and a deep-rooted commitment to animal welfare. Vegans aim to minimize their impact on the environment and reduce animal exploitation by following a plant-based diet and lifestyle. Finally, the term “vegan” has become synonymous with a philosophy that supports a more mindful and conscientious approach to consumption, encouraging individuals to make choices that align with values of compassion and sustainability, hence ethics.

There is also a group of people who follow a raw food diet (raw foodism/fruitarianism) within the vegan philosophy, which has as its core principle the consumption of only fresh, unprocessed fruits and vegetables—avoiding heat-processed foods. This practice seeks to keep their diet as natural and simple as possible and pure [89].

## 7. Food and Spirituality

Food spirituality is defined as “An innate sense of connection that a subject can experience to and through food regarding personal and social identity, culture and ritual nature and the environment, body, and soul, the mundane and the universal”, according to Michopoulou and Jauniškis [90]. Food in religious texts is associated with God’s existence [73]. Spirituality affects our perceptions of everything from mundane to sacred and is overwhelmingly personal and subjective. Life and death beliefs are projected into foods. Is death the end, or does it mark the beginning of a new phase of existence, as posited by Atteshli-Theotoki [31]? Could death merely signify the separation of the physical body, leading to alignment with the psychic or the noetic realm? Atteshli-Theotoki [31] suggests that various scholars, based on her research, view the essence of the self as immortal, referring to the psychic and noetic aspects as the eternal soul. However, this perspective contradicts the findings of White Researchers, who regard the mortal aspect as not only encompassing the physical and etheric bodies but also including elements of the psychic and noetic realms [91]). According to the latter, the true self is associated with the superior spiritual body, often termed the Light Body [31]. Personal spiritual beliefs can be correlated with mental strength, stability, self-control, self-efficacy, and an improved relationship with foods [92,93,94,95]. Improvements in uncontrolled eating, emotional eating, intuitive eating, and mental and spiritual well-being have been shown following a religious program with a spiritual component, as reported by Patel et al. [94,95].

On the other hand, Ayahuasca, a spiritual ritual [96]—also known as the tea, the vine, and la purga—a brew made from the leaves of the *Psychotria viridis* shrub along with the stalks of the *Banisteriopsis caapi* vine, as reported by Savoldi et al. [97], is linked with better emotion control [98], a long-time reduction in depression and stress [99], and the potential to cure drug addiction [100].

A spiritual reconnection of the self to body, nature and society, and eating disorders is strongly discussed, but is it not a spiritual reconnection to God? Why are strong emotions like guilt, shame, well-being, and self-worth alike, or protest and anger suppression, linked with food and eating [90,101] or a feeling of powerlessness and a lack of meaning in life [102,103]? Why is overeating manifested by religious people as a divine struggle or lack of belief [104]?

Overall, religious neighborhoods and communities play a significant role in shaping food choices and practices, as discussed by Tan et al. [105]. However, merely participating in religious ceremonies does not inherently correlate with positive health outcomes [105,106]. Food consumption tends to be more influenced by personal convictions and beliefs rather than strict adherence to religious doctrines [105,106]. Therefore, fostering a deeper connection with God and the Holy Spirit, alongside a strengthening of our faith by our beliefs, appears crucial in this context. A better body–spirit connection, which naturally transfers to better intuitive eating and self-perceived body image, might also be achieved by practicing yoga [107,108].

## 8. Food Dynamics

Globalization, technological advances, changes in consumer preferences, environmental concerns, and socioeconomic disparities affect the dynamics of food in today’s world [29]. Moreover, increased access to a wide variety of foods [109] originates from globalization and the effect of the expansion of food supply chains across different borders worldwide [110]. However, negative impacts are evident due to the vulnerability of systems to disruption, as observed during the COVID-19 pandemic with examples of shortages in some areas and surpluses in others, as reported by Moosavi et al. [111]. Preferences have been largely affected by these disruptions in food systems [112], with consumers looking for alternative food such as organic, plant-based, or locally grown. Consumption of fast and processed foods leads to obesity and diet-related diseases, as discussed and highlighted by Magano et al. [113].

Hence, the compromise of the global food system is considered true and is happening because of a wide range of factors, including the presence of chemical, physical, or microbiological hazards including pathogenic microorganisms, as shown by the WHO [114]. Other reasons involve improper handling and storage; natural toxicity; undeclared allergens; incorrect labeling; animal diseases; exceeded expiry dates; unauthorized genetic manipulation; and fraudulent practices, as elucidated by Fernandez and Paoletti [115]. But we have made this system unsafe because our vibrations of the mind, in the form of thoughts and desires materialized, attract all evil elementals present as inferior forms of life, which plague their creator, Man. It is not God and the Archangels who created mosquitoes, flies, microorganisms, and parasites causing epidemics and abominations, but Man. Man built an unconscious hell for Mankind. This is an example of the Law of Karma or Cause and Effect on a personal, but at the same time, global scale [31]. However, it is essential to recognize the role of human behavior in exacerbating all previous risks. The concept that our thoughts and desires materialize into reality, creating a negative impact on our environment and food systems, has been seriously highlighted by Atteshli-Theotoki [31]. This perspective underscores the interconnectedness of human actions and their consequences, emphasizing the importance of ethical considerations in food production, distribution, and consumption.

To address these challenges and promote a safer and more sustainable food system, stakeholders across the food industry, regulatory bodies, and policymakers must collaborate to implement comprehensive strategies [116]. This may include stringent regulations to ensure that food safety standards are met at every stage of the supply chain, investment in technology and infrastructure to enhance traceability and transparency, support for sustainable agricultural practices, and educational campaigns to raise awareness about the impacts of food choices on health and the environment [117]. Furthermore, enhancing a culture of mindfulness and ethical responsibility among consumers, producers, and policymakers can contribute to the creation of a more harmonious relationship between humanity and the food we consume, aligning with principles of sustainability, social justice, and holistic well-being, as reported by Fallah Shayan et al. [118]. 

## 9. GMOs, Ethics, and Neophobia

Global food governance needs to increase its presence to control questions raised on genetically modified organisms (GMOs), laboratory-grown meat, edible vaccines, and the environmental impact of high-tech agriculture, as discussed by Vega Rodriguez et al. [116]. The ethics or moral dimensions of the GM issue are usually presented in the recent literature as a medley of religious constructs (“playing God”), or moral dilemmas, sometimes mixed with ecological concerns, as reported by Varzakas et al. [4,80]. Playing God is still associated with religion and GMOs [119,120,121].

Cisgenesis and intragenesis were developed as alternatives to transgenesis. Both concepts imply that plants must only be transformed with genetic material derived from the species itself or closely related species capable of sexual hybridization. Furthermore, foreign sequences such as selection genes and vector-backbone sequences should be absent. Allowing the use of new gene combinations created by in vitro rearrangements of functional genetic elements is how intragenesis differs from cisgenesis. Several surveys show higher public acceptance of intragenic/cisgenic crops compared with transgenic crops [122]. One of the major concerns of the public about transgenic crops relates to the mixing of genetic materials between species that cannot hybridize by natural means. Hence, two transformation concepts cisgenesis and intragenesis were developed as alternatives to transgenesis [122,123,124,125].

The presentation of genetically modified produce as “Frankenfoods” [126] is observed as a political myth construct around GM food and GMOs [127]. Posting about the unnatural and artificial sources of food and its association with GMOs and GM food has been widely reported [128,129,130,131,132,133,134]. 

The issue of the unnaturalness of GM food and GMOs has been discussed from different angles such as ethics, nutrition, religion, fear, and safety among other less prominent viewpoints. They all refer to the transgenic nature of GM food and GMOs and conclude that the average consumer cannot relate to an end product that contains traits of two or more organisms that are not naturally (or sexually) compatible; therefore, the term “unnatural” and “artificial” appears in different studies and consultations, without any linkages to the scope of the inquiry of the studies, as reported by Siddiqui et al. [135]. 

Regarding fear, i.e., an emotion that is often expressed in food-related issues, consumer attitudes are shaped by the role of neo-phobia [128,129,130,131,132,133,134,135]. In the case of GMOs, food-related fears affect the behavior of the consumer. Studies in the field show that a lack of information and imbalanced communications constitute the driving force of consumers against GM technology [126,136,137,138,139]. And this has to do with the predominant negative feeling/emotion that is often related to different forms of fear such as fear of isolation, fear of the unknown, fear of consuming products and affecting health, and fear of speaking out on the issue. Specific fears related to GM food and GMOs that are expressed by rivals of GM technology, as reported by Uzogara [140], refer to modification of nutritive quality of foods, possible toxicity, potential allergenicity and potential antibiotic resistance from GM crops, and carcinogenicity from GM food consumption. Furthermore, several concerns refer to other aspects such as environmental contamination, accidental gene transfer to wild plants, potential formation of new viruses and toxins, monopolies in the supply chain of seeds (bio patenting of GMOs), threats to the genetic diversity of plants, and finally religious, cultural and ethical concerns, as well as fear of the unknown. Also, Laros and Steenkamp [126] discussed the frequent appearance of fear messages in the media by examining the Dutch market food market and found that Dutch consumers feel significantly more fearful of GM food than of other new food types. They assumed that there is consistent fear about GM food across society. 

Moreover, neophobia still comprises a niche category among the different types of fear related to GMF; however, this has been overpassed by other fears such as allergens [141]). The display of some degree of human aversion to new foods is a trait named food neophobia, according to Cooke et al. [142]. They added that food neophobia is widespread in omnivores, warblers, rats, and chimpanzees. Knight et al. [143] argue that beliefs about the risks and benefits of the production and introduction of GMF in the food market affect consumer acceptance heavily. Moreover, attitudes towards technology are affected by the associated fears. Finally, many studies refer to “superstition”, “religious fears”, and “magical beliefs”, factors that have been linked with negative attitudes towards GM foods [4,80,144]. 

However, regarding allergens, some studies show that GM crops can be used as a solution to eliminate allergens in food and fight against coeliac disease [145]. One research group developed wheat bread made from wheat flour with a very low gliadin content and the main epitopes of wheat gluten that is potentially suitable for celiac patients and other gluten-intolerant individuals, as discussed by Gil-Humanes et al. [146].

Generally, the ethical dilemmas surrounding GM food and GMOs are multifaceted and complex, encompassing a range of concerns related to human health, environmental impact, and societal values. One of the central ethical dilemmas is the perceived unnaturalness of genetically modified organisms, which raises questions about the integrity of the food supply and the potential risks posed by manipulating the genetic makeup of plants and animals, as noted by Weale [147]. This unnaturalness is often cited as a source of fear and uncertainty among consumers, who may worry about the long-term effects of consuming GM foods on their health and well-being. Additionally, ethical considerations extend to issues such as environmental sustainability and biodiversity, as the widespread adoption of GM crops could lead to unintended consequences such as the emergence of superweeds or the loss of genetic diversity in agricultural ecosystems, as shown by Ghimire et al. [148]. Furthermore, concerns about corporate control and the concentration of power in the hands of biotechnology companies raise questions about equity and justice in the food system, as reported by Fairbairn and Reisman [149]. Addressing these ethical dilemmas requires careful deliberation, transparency, and robust regulatory oversight to ensure that the benefits of GM technology outweigh the potential risks and that the interests of all stakeholders, including consumers, farmers, and the environment, are considered.

## 10. Food Regulation

The debate surrounding the scope of food regulation extends beyond inherently unsafe foods and is influenced by multiple factors, including considerations of public health, ethics, and regulatory efficacy [150]. Proponents of this approach argue in favor of resource efficiency, emphasizing the importance of urgency and adopting a risk-based methodology [151]. Resources can be allocated more effectively when prioritizing regulatory efforts on products with the greatest potential to harm public health. Moreover, adopting a risk-based approach allows for the implementation of stricter regulations on foods with higher associated risks [152]. This targeted strategy enables regulatory bodies to tailor their interventions to address specific threats, thereby enhancing the overall safety of the food supply chain. Such measures not only mitigate risks to public health but also optimize the allocation of regulatory resources, ensuring a more robust and responsive regulatory framework. Arguments against this topic include the complexity of the food system, prevention, and ethical considerations, as discussed by Ververis et al. [153]. The emerging risks or systemic issues in food might be overlooked if there is a limitation of regulation to inherently unsafe foods. Moreover, it should be considered that various sources might cause risks and there might not be confinement of risk to specific foods, as reported by Santeramo and Lamonaca [154].

Achieving a delicate equilibrium between safeguarding public health, upholding individual liberties, and acknowledging cultural and religious diversity is imperative when formulating food regulations for moral or religious reasons [155]. While governmental oversight of food choices plays a crucial role in public health protection, food safety enhancement, and addressing ethical concerns related to food production and consumption [156], it must be executed judiciously. Establishing rigorous standards and robust monitoring systems for food safety across production, distribution, and sale is paramount to ensure the integrity of the food supply chain, as shown by Rose et al. [157]. Between the complexities of government regulation in food and nutrition and competing interests, there is a delicate balance [158]. While it is the government’s responsibility to set nutritional guidelines and standards to safeguard public health, avoidance of excessive regulation that encroaches upon personal autonomy and cultural diversity in food choices should be evident, as highlighted by Little [156]. Moreover, the regulatory landscape is often influenced by powerful commercial interests and companies, which can impede effective governance [159]. Therefore, the adaptability of government authority over food and nutrition should be looked for rather than rigidity [160]). Developing interventions that effectively address public health concerns while respecting the boundaries of government authority is essential for achieving a balanced, ethical, and evidence-based approach, as discussed by Qureshi et al. [161]. It is imperative to establish a dialogue among stakeholders, including policymakers, industry representatives, and community leaders, to devise solutions that promote both public health and individual rights, cultivating a harmonious relationship between regulation and personal freedoms. The regulation of food choices by governments is critical in public health protection, food safety adoption, and addressing ethical issues related to food production and consumption [156]. In this direction, standards and systems must be established, and food safety monitoring should be carried out through regulations for production, distribution, and sale, ensuring the non-threat of food safety, as reported by Rose et al. [157]. The basic legislative forms around the globe are mentioned in Table 1.

## 11. Food Safety and Quality Standards and Ethics

**Figure 1:**
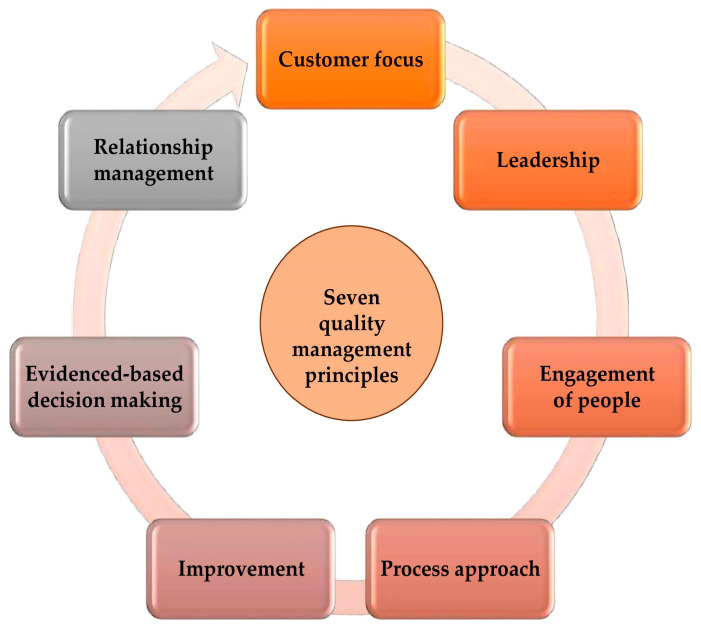
The seven principles of quality management according to ISO 9001:2015 [164] (https://www.iso.org/standard/62085.html (accessed on 12 April 2024)).

Food safety and quality management systems ISO 22000:2018 and ISO 9001:2015 [164] (https://www.iso.org/standard/62085.html (accessed on 12 April 2024)) discuss the issue of ethics from different perspectives. The seven principles of quality management (Figure 1) encompass various aspects such as customer focus, leadership, the engagement of people, the process approach, improvement, evidence-based decision-making, and relationship management. However, these principles may not explicitly address the ethical considerations about human beings and their conduct in the workplace. Ethical leadership and customer care are fundamental aspects that go beyond the scope of quality management principles, focusing on the moral responsibility of leaders and organizations towards their employees and customers [165]. While quality management principles emphasize efficiency and effectiveness in processes and outcomes, ethical leadership and customer care emphasize the importance of integrity, fairness, and empathy in interactions and decision-making [165]. It is then essential for organizations to integrate ethical considerations into their operations, ensuring that employees are treated ethically and customers receive fair and respectful treatment (*The Economist* 2022) [166]. However, all these principles do not deal with human beings and their ethics in a working environment applying ethical principles.

On the other hand, the quality Guru, Deming, described the PDCA (Plan-Do-Check-Act) cycle as a fundamental framework for continuous improvement [167,168,169,170]. Does that cycle not resonate with human beings and their lives? Does it not indicate that before acting ethically and applying our ethical principles, we should effectively plan, implement what has been planned, and then check and monitor accordingly? Is this not the essence of ethics? Why? Are there individuals or organizations that risk not checking monitoring and releasing their products into the market by neglecting these rules? Perhaps there are.

**Figure 2:**
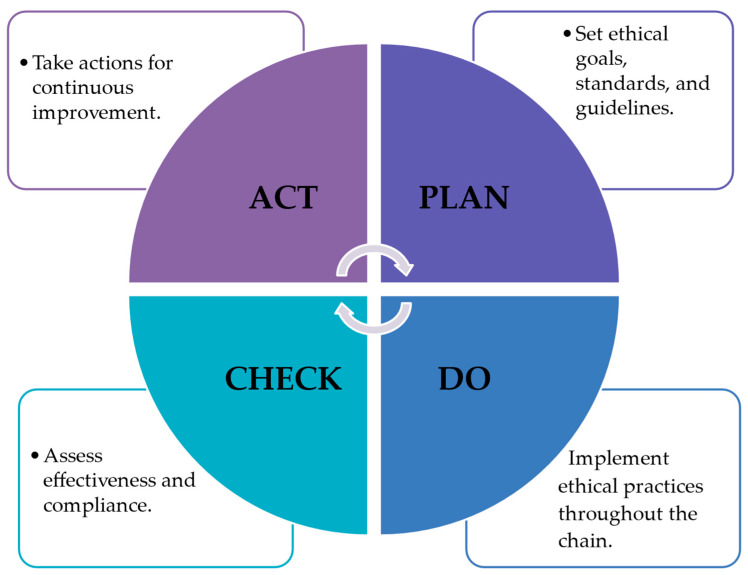
Description of the PCDA cycle in the food chain.

Under this consideration, the PDCA cycle in the food chain should then include the following (Figure 2): 

Plan: In this phase, the focus is on identifying ethical goals and objectives for the food chain. This includes setting standards for sustainable food production, ethical sourcing of ingredients, fair labor practices, and minimizing environmental impact. Stakeholders collaborate to develop policies, procedures, and guidelines that align with ethical principles and regulatory requirements. 

Do: Once the plan is established, it is implemented throughout the food chain. This involves executing ethical practices in food production, distribution, and consumption. Companies may adopt sustainable farming methods, ethical sourcing strategies, and transparent labeling practices. Employees are trained in ethical guidelines, and suppliers are held accountable for adhering to ethical standards. 

Check: In the check phase, the effectiveness of ethical practices is assessed through monitoring, measurement, and evaluation. Key performance indicators (KPIs) are established to track progress towards ethical goals. Audits, inspections, and reviews are conducted to ensure compliance with ethical standards and identify areas for improvement. Feedback from stakeholders, including consumers, is collected and analyzed to gauge satisfaction and identify any ethical concerns. 

Act: Based on the findings from the check phase, actions are taken to address any gaps or deficiencies in ethical performance. This may involve revising policies, updating procedures, providing additional training, or implementing corrective actions. Continuous communication and engagement with stakeholders are essential to drive ongoing improvement and maintain ethical integrity throughout the food chain.

Integrating risk management and risk-based thinking into food ethics is crucial for ensuring the safety, integrity, and ethicality of food production and consumption processes. By applying ISO 31000:2018 principles [171] alongside tools like SWOT analysis, stakeholders in the food industry can identify potential risks to food safety, quality, and ethical standards. This proactive approach allows for the implementation of preventive controls to mitigate risks and seize opportunities that align with ethical values. 

For instance, considering the ethical implications of sourcing ingredients from suppliers with questionable labor practices or environmental sustainability could be part of the risk assessment process. By analyzing strengths, weaknesses, opportunities, and threats, food producers can identify areas where ethical concerns may arise, such as animal welfare, fair labor practices, or ecological sustainability (Leroy et al. 2022) [172]. Implementing preventive measures, such as robust supplier vetting processes or investing in sustainable farming practices, helps minimize negative impacts on ethical standards. Moreover, the concept of risk-based thinking encourages continuous improvement in ethical food practices, as discussed by Thomson [173]. By analyzing nonconformities and their potential ethical implications, organizations can take corrective actions to address underlying issues and prevent recurrence. This proactive approach fosters a culture of ethical responsibility throughout the food supply chain, promoting transparency, accountability, and trust among stakeholders. 

**Figure 3:**
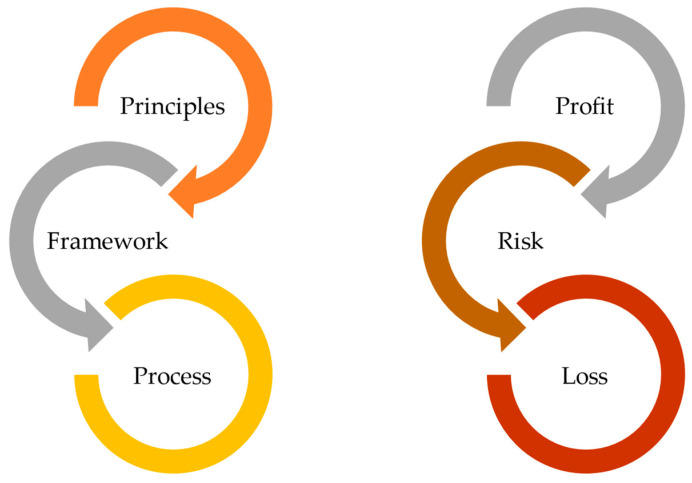
Overview of ISO 31000:2018 (adapted from ISO 31000:2018) [165].

Overall, when we incorporate risk management and risk-based thinking into food ethics, we allow for a systemic approach to identifying, assessing, and addressing ethical concerns in food production and consumption, as described by Ispas et al. [174]. If we proactively manage risks and seize opportunities that align with ethical values, we will allow the food industry to uphold its commitment to ethical standards and contribute to a more sustainable and responsible food system. Managing risk is based on the principles, framework, and process, as reported in ISO 31000:2018 (Figure 3).

**Figure 4:**
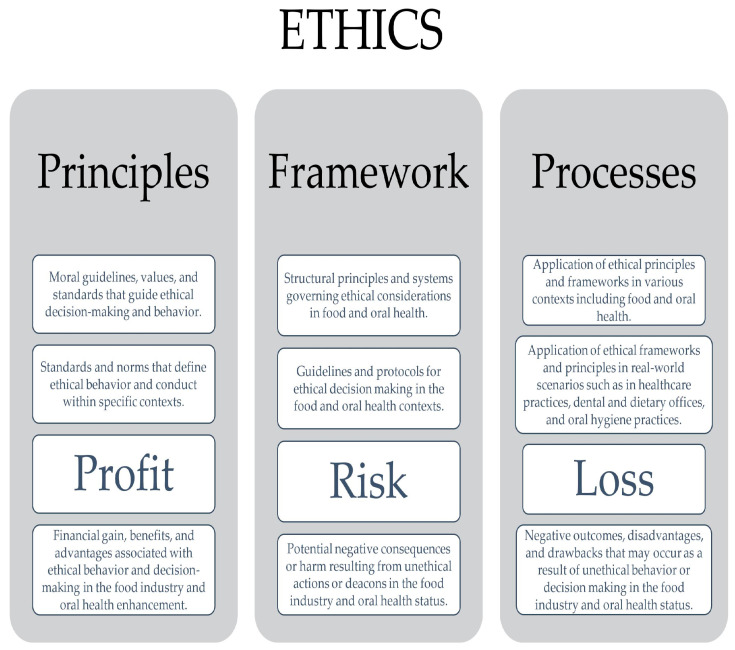
Analysis of ethics according to principles, framework, and processes, as outlined in ISO 31000:2018, in conjunction with profit, risk, and loss; adapted from [165].

Figure 4 illustrates an analysis of ethics in terms of principles, framework, and processes, as outlined in ISO 31000:2018, and connections with profit, risk, and loss [165].

**Figure 5:**
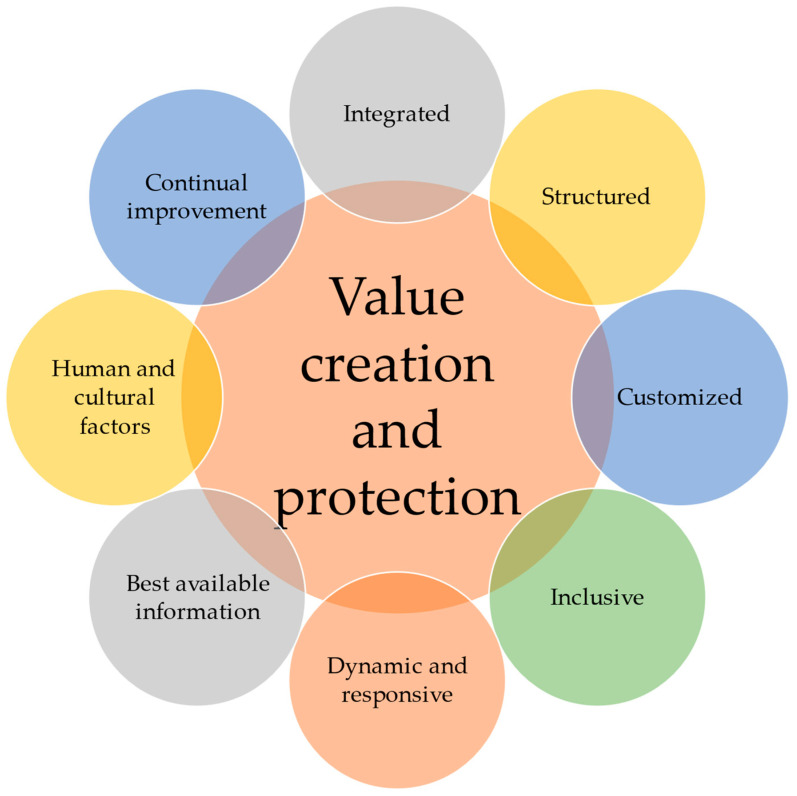
Principles of ISO 31000:2018 (adapted from ISO 31000:2018) [165].

Figure 5 further outlines the principles of value creation and protection (Clause 4), which are paramount in ethical considerations. These principles encompass human and cultural factors, as well as other key elements that drive continual improvement within both individuals and organizations, guided by effective management. 

A synopsis of the ISO principles in the food context is provided in Table 2.

**Figure 6:**
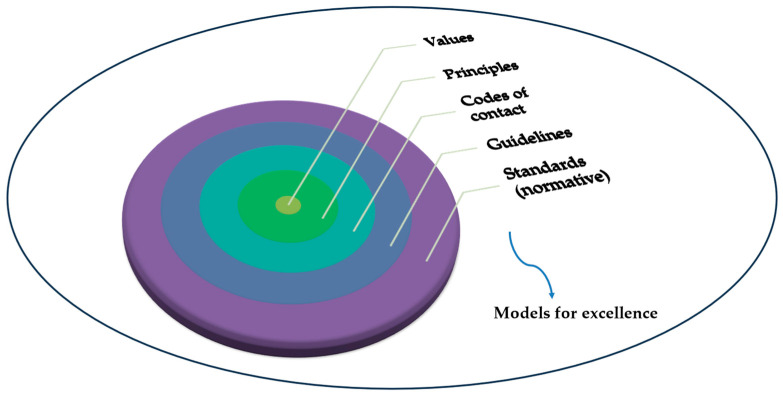
The ripple continuum of standardization.

Figure 6 illustrates the ripple continuum of standardization, depicting how principles and values at the core of these standards influence codes of conduct and models for excellence in the food industry.

**Figure 7:**
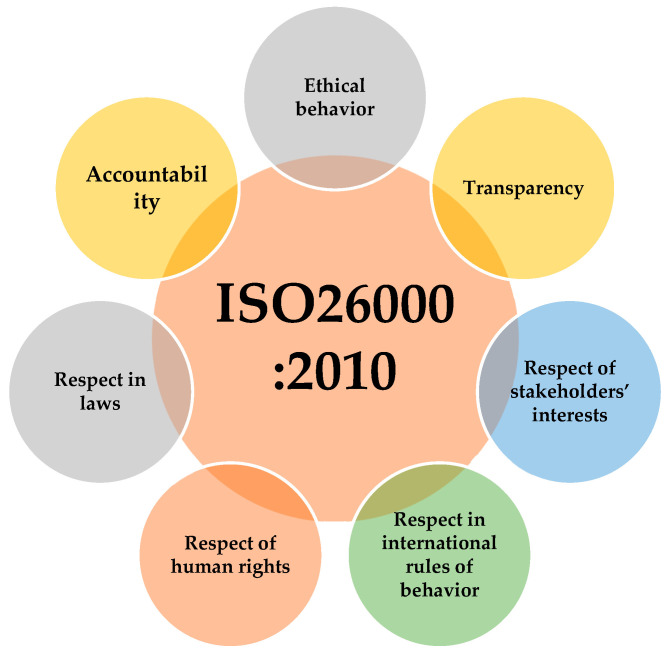
General principles of ISO 26000:2010; adapted from https://www.iso.org/iso-26000-social-responsibility.html, accessed on 12 April 2024.

Furthermore, ISO 26000:2010 provides “guidance on social responsibility”, meaning how businesses and organizations can operate in a socially responsible way and refer to principles of social responsibility related to accountability, transparency, ethical behavior, respect for human rights, respect for stakeholders’ interests, respect for the rule of law, and respect for international norms of behavior. The general principles of ISO 26000 are described in Figure 7.

On the other hand, food safety culture needs to be adopted effectively and requires the implementation of ethics and commitment from the side of people/personnel and management, according to Regulation (ΕU) 2021/382 [175], along with effective leadership and communication from all sides. In this direction, the RSC (Responsible Supply Chain) approach involves responsible sourcing, greater transparency, sustainable practices, and adherence to shared standards and values, as reported by Jacob-John et al. [176]. Amaeshi et al. [177] illustrate the role of the supply chain and the pressure in employing a socially responsible operation. Implementation can be carried out by CSR (Corporate Social Responsibility). Moreover, MacGregor [178] cites five reasons for the implementation of a CSR strategy, and these include risk mitigation, competitive marketing advantage, inter and intra-organizational demands, political–social factors, and conviction.

Corporate responsibility has been broadened to include ethical management, which involves generating economic profits, satisfying the demands of different stakeholders, and ensuring business sustainability. In this context, ethical management has gained prominence as a determinant of business sustainability as it enables organizations to adhere to ethical principles, promote shared social values, and enhance the legitimacy of their operations [179].

The International Organization for Standardization (ISO) released the ISO 26000 standard in 2010 to provide international guidance on social responsibility for all types of organizations. The main objective was to implement CSR by translating the principles and key issues into practical activities and providing best practices in the area of social responsibility. ISO 26000 can be used as a benchmark for assessing the development of the expected relationships of an organization with its environment. Measurable benefits can be brought forward by strengthening stakeholder relationships, enhancing reputation, and building trust [180].

Product-related CSR assesses the management of risk for companies facing significant product recalls or losing customer confidence because of significant product quality concerns. CSR can build a positive reputation and moral capital among stakeholders, mitigating any adverse evaluation of corporate misconduct and ensuring against the potential negative consequences of any violations of stakeholder expectations. A potential product recall is the most common type of disruption with significant consequences for firms in terms of brand reputation and brand equity, as well as financial performance and value [181].

Responsible Supply Chain Management protects consumer confidence and public health while promoting a culture of transparency and ethical behavior in the food industry [176,182]. Through engagement with suppliers, employees, and local communities, RSCM enables companies to establish ethical sourcing practices, empower employees, and champion human rights throughout their supply chains. By promoting ethical labor practices and prioritizing the eradication of modern slavery, RSCM protects vulnerable workers and promotes sustainable and responsible business behavior in the food industry [176,182,183].

Research within the field of responsibility and supply chain management defines responsible behaviors in supply chains in several ways including Green Supply Chain Management, Sustainable Supply Chain Management (SSCM), and Responsible Supply Chain Management (RSCM) [176]. However, it is not only the environmental dimension but, according to Carter and Rogers [184], also the balance between the fiscal, environmental, and social dimensions of the organization. Linnenluecke and Griffiths [185] illustrate that climate change is a major problem that businesses must account for, and climate change results from anthropogenic activities leading to greenhouse gas emissions (GHG). The different perspectives on sustainable food supply are further illustrated by Smith [186] and include food safety and factors supporting the viability and diversity of the stakeholder economics and communities along with consideration of the ecological impacts. According to Smith [186], ecological aspects of food involve environmental issues such as a reduction in energy consumption and minimization of water use, whereas social aspects involve the creation and maintenance of a safe and socially acceptable working environment for all employees across the entire supply chain considering all stakeholders. 

Overall, various ISO standards are relevant to ethical actions and practices in the food industry, covering areas such as social responsibility, food safety, environmental management, quality management, risk management, occupational health and safety, sustainable procurement, energy management, business continuity, information security, and anti-bribery measures. The most important ones are described in Table 3.

## 12. Food Fraud

Wognum et al. [187] state that transparency is pivotal for sustainable operations within food supply chains. Better traceability and transparency within supply chains arose in 2013 with the horsemeat scandal in Europe. According to Rasul and Thapa [188], the increasing and incessant use of chemicals during farming processes by using fertilizers, insecticides, and pesticides not only affects the environment negatively with the increase in maximum residue limits (MRLs) but also the society by spreading diseases to humans and affecting aquatic life and livestock.

Spink and Moyer [189] define food fraud as all intentional acts that involve deliberate and intentional substitution, addition, tampering, misrepresentation, or false/misleading statements performed to gain economically [190,191,192,193,194,195,196,197,198,199,200,201]. This poses three types of risks—direct, indirect, and technical risks. 

Rampant mislabeling is also connected with fraud. The case of New Zealand Manuka honey is well known [202,203]. Rampant use of other adulterants including formalin, urea, starch, neutralizers, detergents, sodium chloride, skim milk powder, sucrose, glucose/dextrose, and hydrogen peroxide [204,205]. It is a clear action of producers’ profit through misconduct and unethical behavior versus the expense of the health of the consumer [206].

Moreover, Fassam et al. [207] reported on four supply chain drivers for food fraud including lack of trust among supply chain actors, opportunistic behaviors by supply chain partners, inadequate governance of the supply chain, and complexity of the supply chain. Niu et al. [208] further investigated the food fraud key influencing factors and their interrelationships in an emerging food market—China—by using the DEMATEL-based analytic network process (DANP) and showed that the identified key cluster was government regulation, social governance, and detection techniques. A summary of food fraud types and ways that consumers may acknowledge them is provided in Table 4.

## 13. Consumer Behavior and Ethics

Consumers’ beliefs and intentions such as health values and consciousness have been linked and interrelated with organic food purchase and consumption preferences as reported by many researchers [209,210,211,212,213,214], nutrition content [210,215,216], environmental concern [217,218,219,220,221], safety [222,223,224] and taste [225,226,227]. Consumer awareness about food scandals, epidemics of diseases from viruses such as Bovine Spongiform Encephalopathy or pathogenic bacteria such as *Escherichia coli* 0157 infections, and favorable weather patterns [210,228,229] has also been reported widely.

Guru et al. [230] analyzed the motivational factors critical to the purchase and consumption of organic food and extended a roadmap to the food industries for sustainable growth. They found that the purchase and consumption of organic food products are affected by issues such as being chemical-free, having no artificial ingredients, being good for the body, being healthy, and having an awareness of health. 

The growing interest in fresh, healthy, and organic agriculture and food products has also been elucidated by many researchers [231,232,233]. Furthermore, health concerns, religious beliefs, environmental concerns, animal welfare, human rights, fair trade, and fair wages are considered by consumers in food consumption behavior [234,235]. Hence, food ethics plays an important role in understanding consumers’ perceptions [236,237]. Overall, the general theory of marketing ethics developed by Hunt and Vitell [238] states that a person’s deontological evaluation (DE) and teleological evaluation (TE) are related to his/her ethical judgment [235]. In this direction, DE and TE significantly affected positively perceived behavioral control and subjective norms, as reported by Ho et al. [239].

## 14. The Mouth—The Sacred Gateway to the Body

The phrase “the mouth is the gateway to the whole body” encapsulates a profound understanding of the interconnectedness between oral health and overall well-being, resonating throughout ancient wisdom, biblical teachings, contemporary scientific research, and holistic health principles [10,240,241]. In ancient civilizations such as Greece and Rome, this concept finds resonance in the interplay between oral health and bodily equilibrium [242,243,244]. Rooted in the humoral theory described by Hippocrates, oral diseases were viewed as manifestations of disharmony within the body, stemming from imbalances in bodily humor, as reported by Bujalkova et al. [245]. For ancient Greek philosophers, the mouth served not only as a site of ingestion but also as a conduit through which imbalances in diet and environment could influence systemic health [246,247]. The classification of foods based on inherent qualities and seasonal variations in dietary recommendations underscored the recognition of the mouth’s pivotal role in maintaining humoral balance and preventing disease [245]. This holistic understanding suggests that oral health is not an isolated entity but rather intricately linked to the well-being of the entire body, emphasizing the importance of reviewing oral health as a cornerstone of holistic health practices across cultures and periods [12].

Seasonal dietary variations and mouth intakes, crucial for humoral balance and disease prevention, were acknowledged in ancient medical practices too [245,247]. In Hippocratic writings such as “*Airs, Waters, and Places*”, seasonal changes were recognized as influencing disease patterns, highlighting the impact of environmental factors on health [246]. Consequently, dietary recommendations varied with the seasons, with specific foods prescribed to counteract seasonal imbalances, reflecting a holistic approach to health that emphasized the interconnectedness of diet, environment, and overall well-being [248,249,250]. The Hippocratic treatise “*On Regimen*” epitomizes the fusion of philosophy and dietetics, providing us with a balanced approach to health authored by the Hippocratic writers [251]. This work focuses on moderation and temperance in dietary habits, stressing the fundamental role of food in maintaining bodily equilibrium [251]. Central to Hippocratic philosophy is the belief that food serves as a cornerstone of well-being, resonating with the ancient Greek concept of harmony in life. Anaxagoras of Clazomenae, a pre-Socratic philosopher, contributes to this discourse through his fragmentary teachings. Curd (2007) [252] presents Anaxagoras as a thinker deeply concerned with the nature of the cosmos, yet his insights extend to the realm of nutrition. Anaxagoras proposes that food sustains life by providing nourishment to the body, aligning with his broader cosmological theory that all things are composed of infinitesimally small particles known as “nous” or mind (Curd 2015) [253]. In this context, the act of eating transcends mere sustenance; it becomes a reflection of the cosmic order, wherein the ingestion of food symbolizes the assimilation of universal principles into the individual. Pythagoras, also renowned for his mathematical and metaphysical teachings, espoused a distinct dietary philosophy [254]. Dye (1999) [255] explores the enigmatic prohibition of beans in Pythagorean doctrine, attributing it to symbolic and practical reasons. For Pythagoras, beans symbolized impurity and were associated with death rites, thereby conflicting with his belief in the transmigration of souls [256]. According to this theory, the Pythagoreans believed that all living beings, plants, animals, humans, vampires, ninja turtles, etc., shared a common soul or life force that could be reborn in different forms after death, as noted by Huffman [257]. In this context, beans may have been seen as particularly problematic, as they were believed to contain the souls of the dead. By avoiding beans, the Pythagoreans may have believed that they could avoid being contaminated by any potentially impure or malevolent spirits. It seems that beans were/are what you incarnate as if you have been a bad human. Moreover, Pythagoras suggested a diet free from animal products, viewing vegetarianism as cultivating moral and spiritual purity [258]. Through dietary restrictions, Pythagoras sought to align the physical body with the principles of harmony and ethical integrity, as discussed by Graham [259].

The philosophical inquiry into the mouth and food extends beyond individual thinkers to encompass broader cultural practices and beliefs [260]. Garnsey [261] examines the connection between food and society in classical antiquity, shedding light on how dietary customs reflected social hierarchies, religious beliefs, and agricultural practices. From the extravagant banquets of the elite to the simple fare of the common populace, food served as a marker of identity and status in ancient Greek society, as shown by Flint-Hamilton [262]. Furthermore, the philosophical contemplation of food intertwines with religious rituals and mysteries. Delatte [263] set light on the mystical significance of the “kykeon”, a barley-based beverage consumed as part of the Eleusinian Mysteries. This sacred concoction symbolized nourishment for the soul through the mouth, offering initiates a transformative experience of spiritual enlightenment. The consumption of kykeon exemplifies how food transcends its material essence to become a conduit for metaphysical experiences and revelations [264]. As it seems, the mouth was considered by the ancients as “the gateway to the body”, which encompasses notions of health, morality, cosmology, and spirituality that enhance the profound significance of sustenance and sustainability in shaping individual and collective well-being [265]. 

Central to this exploration is the passage from Matthew 15:10-1 4 [266], wherein Jesus challenges conventional understandings of purity and underscores the primacy of inner purity over external rituals, shedding light on the profound spiritual dimensions of oral health. Jesus’ teachings within this passage challenge the conventional understanding of purity, urging individuals to see beyond mere external rituals and embrace the transformative power of inner purity. He elucidates that true defilement arises not from external contaminants but from the inner recesses of one’s heart, emphasizing the profound link between spiritual purity, food intake, and physical well-being. This paradigm shift redirects attention to the status between one’s internal disposition and external health outcomes, highlighting the holistic nature of human existence wherein spiritual and physical domains intersect [267]). Through this lens, oral health emerges not merely as a matter of hygiene but as a reflection of one’s spiritual alignment with inner and outer purity. As Hawks [267] further states, this perspective invites us to recognize oral health not merely as a physical concern but as a reflection of our spiritual well-being, highlighting the transformative power of inner purity in fostering holistic health. Furthermore, Chan et al. [268] elaborate on a body–mind–spirit model in health, proposing a comprehensive approach that integrates spiritual well-being with physical and mental health. Moreover, research by Spanemberg et al. [269] and Fiorillo [270] demonstrates the profound impact of oral health on overall quality of life, highlighting its pivotal role in pursuing holistic well-being. This holistic perspective is further supported by studies on oral health-related quality of life among diverse populations, including older adults [271,272], older adults receiving home health care services [272], institutionalized residents [273,274], children and adolescents [275], individuals of varying socioeconomic status [276], and postpartum women [277]. 

## 15. Significance of Oral Health in the Food Chain 

The mouth serves as a crucial gateway to the body because of its pivotal role in multiple physiological processes and its direct connection to various systemic health conditions such as digestion, respiration, pathogen defense, systemic health, and communication, as reported by Kazemi et al. [278]. First and foremost, the mouth is where the process of digestion begins. Chewing, or mastication, breaks down food into smaller particles, facilitating digestion and nutrient absorption further along the digestive tract [279]. Additionally, the mouth contains salivary glands that produce saliva, which not only moistens food to aid in swallowing but also contains enzymes that initiate the breakdown of carbohydrates (Pedersen et al. [280]). Beyond its digestive functions, the mouth is intricately connected to the respiratory system through the oral and nasal cavities. Proper breathing relies on unobstructed airflow through the mouth and nose, highlighting the mouth’s role in oxygen intake and gas exchange [281]. Moreover, the mouth serves as a primary entry point for pathogens, bacteria, and foreign substances through food consumption and individual ways of function. Its warm and moist environment provides an ideal breeding ground for microorganisms, making oral hygiene crucial for preventing infections and maintaining overall health (Deo and Deshmukh 2019) [282]. 

Poor oral hygiene can lead to various oral health issues such as dental caries, periodontal diseases, and oral infections, which can have systemic implications, as shown by Bhatnagar [283]. Diseases of the oral cavity affect approximately 50% of the global population, which accounts for around 3.5 billion people, making them the most prevalent health condition worldwide. Among the most widespread oral diseases are untreated dental caries of both deciduous and permanent teeth, severe periodontal disease, oral candidiasis, and oral cancer (WHO, [284]). Oral health issues can have significant implications for individuals, including pain, discomfort, difficulty eating and speaking, and in severe cases, even life-threatening conditions such as oral cancer, as reported by Barranca-Enríquez and Romo-González [285]. Moreover, poor oral health can impact overall well-being, leading to social and psychological consequences, reduced quality of life, and increased healthcare costs, as highlighted by de Abreu et al. [286]. 

For these reasons, the connection between oral health and systemic health has been the scope of serious research investigations in the last two decades [287]). Oral health has been associated with various systemic conditions, such as cardiovascular disease, diabetes, respiratory infections, adverse pregnancy outcomes, high blood pressure, pulmonary diseases, low birth weight, Alzheimer’s disease, osteoporosis, and rheumatoid arthritis [288]. Periodontal disease, characterized by inflammation and infection of the gums and supporting structures of the teeth, has been identified as a potential risk factor for the development and progression of systemic diseases such as cardiovascular disease, diabetes, and cancer [289]). Romandini et al. (2021), [290] reported that individuals with periodontal disease are 3.11 times more likely to experience mortality from stroke, 2.58 times more likely from cardiovascular disease, 1.67 times more likely from diabetes, and 1.38 times more likely from cancer. Such reports highlight the significant association between periodontal disease and various systemic conditions, indicating that oral health is closely linked to overall health outcomes [291]). This connection extends beyond dental hygiene practices to encompass broader lifestyle factors, including dietary habits. Poor dietary choices, such as high sugar intake and low nutrient consumption, can contribute to the development and progression of periodontal disease and dental caries, as reported by Dimopoulou et al. [292]. Sugary foods and beverages promote the growth of harmful bacteria in the mouth, leading to plaque formation and gum inflammation, as reported also by Antoniadou and Varzakas [138] and Pang et al. [293]. Additionally, a lack of essential nutrients, such as vitamins C and D, calcium, and antioxidants, compromises the body’s ability to fight oral infections and maintain healthy gum tissue [294]). Conversely, a balanced diet rich in fruits, vegetables, lean proteins, and whole grains can support oral health by providing essential nutrients and antioxidants [295,296]. For example, foods high in vitamin C, such as citrus fruits and leafy greens, promote gum healing and reduce inflammation. Dairy products fortified with calcium and vitamin D contribute to strong teeth and bones, while lean proteins facilitate tissue repair and maintenance [138,297]. Therefore, promoting a nutritious diet as part of oral health education and preventive care initiatives is essential for reducing the incidence and severity of periodontal disease. 

Moreover, dental caries, primarily caused by the consumption of free sugars, is a prevalent and costly disease with significant implications for general health and quality of life [298]. It was and still is a major public health problem globally and is the most widespread noncommunicable disease (NCD) [299,300]. It is also the most prevalent condition included in the 2015 Global Burden of Disease Study, ranking first for the decay of permanent teeth (2.3 billion people) and twelfth for deciduous teeth (560 million children) [301]. Dental caries is a leading reason for tooth extraction, which can exacerbate chronic pain and contribute to systemic infections [302]. Moreover, severe dental caries disproportionately affects vulnerable populations, including children and those in low- and middle-income countries, highlighting the need for accessible preventive and treatment measures [303]. Sugar-sweetened beverages, such as fruit-based and milk-based drinks, along with 100% fruit juices, confectioneries, cakes, biscuits, sweetened cereals, desserts, sucrose, honey, syrups, and preserves, serve as primary sources of free sugars in many countries, putting the base for the expansion of the disease [298]. To control the risk of dental caries across all stages of life, it is recommended to limit free sugar intake to less than 10% of total energy intake [292]. Ideally, reducing this intake further to less than 5% offers additional protection against dental caries [304].

If healthcare professionals emphasize the importance of dietary choices in maintaining oral hygiene and overall health, individuals can take proactive steps to safeguard against systemic conditions linked to poor oral health, such as cardiovascular disease, diabetes, and cancer [292]. This is the first step to a wide interdisciplinary approach to controlling oral diseases and other systematic ones that are reducing the sustainability of the human ecosystem [286,288,305]. Recent findings underscore the importance of maintaining good oral hygiene and seeking timely treatment for periodontal disease to reduce the risk of adverse health outcomes [306]. Moreover, they emphasize the need for interdisciplinary collaboration between dental and medical professionals to address the bidirectional relationship between oral health and systemic health effectively [307]. Early detection and management of periodontal disease may contribute to improving overall health and reducing the burden of chronic diseases globally, and the prevention of dental caries may increase quality-adjusted life expectancy [306,308,309]. 

All relevant data highlight the mouth’s significance as a potential site for the transmission of infections and inflammation to other parts of the body [310,311,312]. More specifically, the study of the oral microbiome has increasingly unveiled its critical role in both oral and systemic diseases, shedding light on complex interactions within the human body [310,313]. Recent research, such as that conducted by Peng et al. [310], has emphasized the significance of oral microbiota in systemic diseases, highlighting the interconnectedness between oral health and overall well-being. So, understanding the fundamentals of the oral microbiome has become essential for oral healthcare professionals [313,314,315]. Moreover, investigations into bacteriophages in the oral cavity have revealed their potential roles in oral health and disease [316,317]. This microbiome complexity extends beyond the oral cavity, influencing various systemic conditions such as inflammatory bowel disease [318,319,320], non-alcoholic fatty liver disease [321,322,323], diabetes [324,325,326], and even neurodegenerative disorders like Alzheimer’s and Parkinson’s disease [327,328,329]. Additionally, associations have been found between the oral microbiome and conditions like depression, anxiety, and suicidal ideation [330,331,332,333] and cardiac diseases [334,335]. The implications of the oral microbiome extend to reproductive health, with evidence linking it to adverse pregnancy outcomes [336,337,338], while it has been reported that periodontal treatment improves prostate symptoms and lowers serum PSA in men with high PSA and chronic periodontitis [339]. Furthermore, its involvement in autoimmune diseases [340,341], autoimmune hepatitis [342], bowel disease [343], respiratory conditions [344,345], and even cancer development [346,347,348] underscores its systemic impact. Saliva, as a readily accessible diagnostic medium, has emerged as a valuable tool in understanding oral and systemic health [349] and body composition in early childhood [350], with ongoing research focusing on its potential for point-of-care testing and disease monitoring [351,352].

Furthermore, the mouth plays a vital role in communication and expression, serving as the primary organ for speech and articulation. The tongue, lips, and palate work together to produce sounds and convey meaning through language, facilitating social interaction and emotional expression [353]. Any disruptions or abnormalities in oral structures can impact speech clarity and communication abilities. Maintaining good oral hygiene and addressing oral health issues promptly is essential for overall well-being and systemic health [354]. Serious work has been performed also on the impact of tooth loss and edentulism on the quality of life of affected individuals [355,356]. Edentulism, the condition of being without teeth, can have profound physical, psychological, and social consequences, significantly affecting an individual’s overall well-being [357]. There are multiple challenges faced by patients with edentulism, including difficulties in eating and speaking, compromised aesthetics, and decreased self-esteem. These challenges can lead to social isolation, anxiety, and depression, further exacerbating the negative impact on quality of life [138,295]. It is suggested that the needs of edentulous patients should be addressed through comprehensive oral healthcare interventions [138,357]. This includes providing access to dental prostheses, such as dentures, to restore oral function and aesthetics. Additionally, psychological support and counseling may be necessary to help patients cope with the emotional and social consequences of tooth loss. In Table 5, the significance of oral health in the food chain is presented.

The above synoptic report suggests that oral health stands as a fundamental component of holistic well-being, encompassing both physical and spiritual dimensions, and serves as the gateway to overall health [284,358]. 

## 16. Economic Implications and Ethics in the Prevention and/or Provision of Oral Health 

The economic implications of oral health extend far beyond individual well-being to encompass broader societal and financial dimensions [359,360]. Listl et al. (2015) [361]. highlight the substantial economic burden of oral diseases, including direct treatment costs, productivity losses from missed school and workdays, and diminished quality of life. In 2015 alone, dental diseases worldwide amounted to approximately USD 545 billion in total costs, with significant direct and indirect expenses [362]. Particularly in high-income countries like the United States, oral health issues result in considerable productivity losses comparable to those associated with musculoskeletal disorders [363,364,365,366]. Moreover, oral diseases exacerbate the impacts of other conditions such as diabetes, yet effective periodontal treatment has been shown to reduce overall healthcare costs [367]. Importantly, out-of-pocket dental expenses can push economically vulnerable families towards poverty [368], while inadequate access to dental care contributes to the inappropriate use of emergency departments and physician offices [369,370,371]. 

The study conducted by Jeffcoatt et al. (2014), [372] sheds light on the significant economic benefits that could be achieved by addressing periodontal disease in patients with systemic conditions such as diabetes, cardiovascular diseases, and a history of stroke. According to their findings, treating periodontal disease could result in a substantial annual economic benefit for patients with these conditions, including a reduction in healthcare utilization by 40.2% for diabetic patients, 10.7% for patients with cardiovascular diseases, and 40.9% for patients with a history of stroke. Moreover, there could be a 67% decrease in the need for hospitalization and a 54% decrease in the need for emergency care, leading to a lighter economic burden on the healthcare system.

The implications of these findings are profound, as they suggest that investing in periodontal treatment not only improves oral health but also contributes to overall systemic health and reduces healthcare costs. Avalare Health LLC, based in Washington DC [373], projected that the expenses for periodontal treatment from 2016 to 2025 would amount to USD 7.2 billion. However, by reducing healthcare expenses related to systemic diseases by USD 70.7 billion, there could be a net gain of USD 63.5 billion. This surplus could be directed towards enhancing healthcare infrastructure, hospitals, equipment, and staffing, as well as investing in wellness initiatives and resilience-building for both patients and healthcare systems. We can imagine the amounts saved if we could provide even more preventive care for dental caries control worldwide, which is the most prevalent disease of all (Heng 2016; NIH; Kruk et al. 2018) [359,374,375].

On this issue, the WHO’s Global Oral Health Status Report [376] provides a comprehensive overview of oral disease burden and serves as a reference for policymakers and stakeholders to prioritize oral health on global, regional, and national agendas. The WHO 2021 proposal [377] on oral health aims to address the global burden of dental diseases through a multifaceted approach. It emphasizes preventive measures, such as regular dental check-ups, fluoride treatments, and oral hygiene education for both caries and periodontal disease control, to reduce the prevalence of dental issues. Universal access to essential oral healthcare services is advocated for ensuring equitable access for all individuals regardless of socioeconomic status. The integration of oral health services into primary healthcare systems is promoted to deliver holistic healthcare. Community water fluoridation programs are encouraged for their effectiveness in preventing dental caries. Health promotion strategies are highlighted to raise awareness about oral health and encourage healthy behaviors. Continuous data collection and monitoring systems are also necessary to track oral health indicators and guide policy development [378]. Collaboration among governments, healthcare providers, NGOs, and other stakeholders is emphasized to implement comprehensive oral health strategies effectively. Finally, strict decision-making processes by international organizations should be followed urgently by actions globally and nationally [379].

## 17. Sustainability Issues in the Food Chain until the Mouth Gateway

The intersection of sustainability, oral health, and food ethics is an area of growing interest and importance. Sustainable practices in oral healthcare sectors involve not only addressing the immediate needs of patients but also considering the long-term implications for both individual health and the environment. For example, promoting preventive measures such as regular dental check-ups and oral hygiene education not only prevents oral diseases but also reduces the need for more invasive and resource-intensive treatments in the future [376]. Additionally, adopting sustainable practices in dental clinics, such as minimizing waste and using eco-friendly materials, contributes to environmental conservation efforts. 

Furthermore, food ethics play a crucial role in oral health and overall well-being. A diet rich in nutritious foods not only supports oral health by providing essential nutrients for gum and tooth health but also promotes systemic health and reduces the risk of chronic diseases (Clemente-Suárez et al. 2023; Kalpe et al. 2023) [296,380]. Encouraging sustainable food choices, such as locally sourced and organic produce, not only benefits individual health but also supports ethical food production practices and reduces the carbon footprint associated with food transportation and processing (van Bussel et al. 2022) [381]. We could also enhance periodontal and oral health, enforcing research to evaluate the clinical, microbiological, and immunological effects of probiotic supplementation and other food derivatives for preventing and treating periodontal diseases (Gheisary et al. 2022) [382] and dental caries (Voidarou et al. 2022) [383]. Finally, we could suggest further research into the development and characterization of an oral microbiome transplant based on food extracts as a novel treatment approach for dental caries and periodontal disease (Nath et al. (2021)) [384].

In terms of legislation and information-sharing initiatives, there is a pressing need for comprehensive policies that recognize the intricate relationship between oral health, systemic health, and sustainability. Legislative measures should aim to incentivize preventive dental care, advocate for community water fluoridation, and implement regulations to minimize the environmental footprint of dental practices. Concurrently, information-sharing initiatives ought to focus on raising awareness about the pivotal role of oral health in overall systemic well-being, promoting sustainable oral healthcare practices in both healthcare professionals and the public, and fostering collaboration between dental and medical sectors to deliver holistic patient care (Glick et al. 2023; Fisher et al. 2023) [385,386]. Addressing oral diseases not only enhances oral health outcomes but also brings about substantial economic benefits and contributes to broader systemic health and sustainability goals (van Bussel et al. 2022) [381]. Investing in preventive oral healthcare practices, advocating for sustainable dietary choices, and implementing legislative and information-sharing initiatives is the way to collectively forge a healthier and more sustainable future for individuals, communities, and the planet. The significant economic advantages of improving oral health and ensuring equitable access to dental care underscore the critical importance of addressing oral health disparities and inequities within communities (Bhatnagar, 2021; Kruk 2021) [358,359].

Reflecting on over 2500 years since Hippocrates articulated his four pillars of medical ethics (Jhala and Jhala 2012) [387], it is evident that these principles continue to serve as fundamental guides for medical practitioners worldwide. The enduring relevance of “Primum non nocere”, emphasizing the duty to avoid harm, “Beneficence”, urging action in the patient’s best interest, “Autonomy”, recognizing patient self-determination, and “Justice”, ensuring equitable healthcare, highlights their timeless significance in ethical medical practice. However, as medical knowledge and technology progress, challenges emerge in interpreting and applying these principles, particularly in areas such as nutritional support and end-of-life care (Jones, 2010) [388]. The ethical dilemma of respecting patients’ wishes regarding nutrition, especially in cases of cognitive impairment or terminal illness, underscores the importance of careful ethical decision-making processes that prioritize patient well-being while acknowledging medical limitations (Cardenas 2021) [389]. Embracing a holistic approach to food consumption, individuals can honor the sanctity of life through mindful selection and consumption, acknowledging the interconnectedness of dietary choices, environmental impact, and personal well-being [390]. In such a case, the quality and quantity of what comes in through the mouth concerning the energy profile represent what is expected to come out in the form of health quality, expression, communication quality, positive choices and actions, exceptional energy performance, and ethical decisions, as there seems to be a fundamental role of sensory experiences in shaping our ethical beliefs and behaviors [391]). Overall, by honoring life through mindful oral practices and food choices, individuals not only contribute to a more ethical and sustainable food ecosystem but also nurture their health and connection with the world around them, providing tools for sustainability for the human ecosystem. Christ has urged us to be unified, echoing the sentiment of “that they may be one” (John 17:21), signifying the interconnectedness and unity we should strive for with our Creator, ourselves, and our fellow citizens.

## 18. Limitations of the Study

This study possesses some limitations that need consideration. Firstly, its non-systematic approach to a literature review introduces the risk of bias in the selection and interpretation of studies. Without adhering to a structured methodology, there is a possibility of overlooking key literature, leading to a potential lack of comprehensiveness in coverage. Additionally, the broad scope of the topic may hinder the depth of analysis, potentially resulting in a superficial exploration of certain complex issues. Furthermore, the inclusion of literature may be limited by factors such as availability, accessibility, and language barriers, particularly from regions with limited research infrastructure. Consequently, there is a risk of representation bias, where certain perspectives or experiences are underrepresented. Moreover, the interpretation of findings may be influenced by subjective biases, impacting the objectivity and validity of conclusions drawn. Despite these limitations, transparent reporting and cautious interpretation have mainly diminished these concerns and enhanced this study’s credibility.

## 19. Areas for Further Research

A future perspective of this study could be conducting longitudinal studies to assess the long-term impact of legislative interventions, such as the implementation of food safety laws or sustainability initiatives, which can provide valuable insights into their effectiveness over time. Additionally, investigating the socio-cultural factors influencing food-related behaviors and ethical decision-making across diverse populations and regions can offer a more extended understanding of ethical dilemmas in food consumption and production. Furthermore, examining the role of emerging technologies, such as blockchain or artificial intelligence, in enhancing transparency and accountability within the food supply chain might contribute to more robust ethical frameworks. Exploring the intersectionality of food ethics with other disciplines, such as public health, economics, and environmental science, can also shed light on interconnected challenges and opportunities for holistic solutions across the globe. Moreover, studying the ethical implications of alternative food production methods, such as lab-grown meat or insect protein, can inform debates surrounding sustainability and animal welfare. Lastly, investigating the relationship between food ethics, spirituality, and well-being can offer novel perspectives on the ethical dimensions of food consumption and its broader implications for human flourishing. Addressing these future research directions may help scholars contribute to the development of evidence-based policies and practices that promote ethical and sustainable food systems globally.

## 20. Conclusions

Ethical decisions in the food chain not only contribute to preventing food fraud and ensuring the safety and integrity of the food supply but also have broader implications for global health and well-being. Societies can foster healthier communities and promote social justice if they allocate resources saved from combating food fraud and unethical practices towards addressing hunger, controlling oral diseases, and managing systemic health issues. Moreover, ethical behavior reflects a sense of responsibility towards oneself, others, and the greater good, aligning with moral and spiritual values that emphasize compassion and care for all living beings. In essence, being ethical in the food chain encompasses not only personal integrity but also a commitment to promoting human dignity and honoring the interconnectedness of all life, thereby reflecting reverence for the divine creator.

## Figures and Tables

**Table 1:** Comparison of food legislation around the world [162,163] (https://food.ec.europa.eu/horizontal-topics/general-food-law_en) (accessed on 12 April 2024), https://food.ec.europa.eu/safety/biological-safety/food-hygiene_en (accessed on 12 April 2024).

Region	Legislation	Key Points
United States	Food Safety Modernization Act (FSMA)	Focuses on the prevention of foodborne illnesses
	Federal Food, Drug, and Cosmetic Act	Regulates food safety, labeling, and additives
European Union	General Food Law Regulation	Ensures food safety, traceability, and labeling
	Regulation (EC) No. 178/2002	Establishes general principles and requirements of food law
	Regulation (EU) No. 1169/2011	Deals with food information provided to consumers
	European Union Regulation (EC) No. 852/2004, 853/2004, 854/2004, 882/2004, Regulation (EU) 2017/625	Sets hygiene rules for foodstuffs in the EU
Scandinavian countries	Norway Food Act No. 124 of 2003	Regulates food safety and quality in Norway
	Sweden Food Act (2006:804).	Sets requirements for food safety and labeling in Sweden
	Finland Food Act (297/2021)	Regulates food production and safety in Finland
	Denmark Food Act (No. 46 of 2017).	Ensures food safety and quality standards in Denmark
Germany	German Food and Feed Code (LFGB)	Regulates food safety and quality in Germany
	German Food Hygiene Regulation (LMHV)	Establishes hygiene requirements for food businesses
Africa	African Union Model Law on Food Safety	Aims to harmonize food safety laws across African countries
	Tanzania Food, Drugs and Cosmetics Act, 2003	Regulates food safety, drugs, and cosmetics in Tanzania
Middle East	Gulf Cooperation Council (GCC) Food Law	Establishes food safety regulations in GCC countries
	Saudi Arabia Food Law (Royal Decree No. M/1), 30 October 2014	Regulates food safety standards and practices in Saudi Arabia
	UAE Federal Law No. 10 of 2015 on Food Safety	Focuses on ensuring food safety and quality in the UAE
Japan	Food Sanitation Act	Regulates food safety and hygiene in Japan
Asia	ASEAN Guidelines on Food Hygiene	Provides guidelines for food safety and hygiene in ASEAN countries
	Food Safety Act (Republic of Korea) (Consolidated version of Act No. 9432 of 2009 as amended last by Act No. 18967, 10 June 2022)	Regulates food safety standards in South Korea
	Food Safety Act (Taiwan) Act Governing Food Safety and Sanitation (“Act”), last amended on 24 January 2018	Ensures food safety and quality standards in Taiwan
	Food Safety Act (Singapore) Food (Amendment) Regulations 2023, 17 April 2023	Regulates food safety and hygiene in Singapore

**Table 2:** Ethical principles for value creation and protection in the food industry ISO 31000:2018, [165].

Ethical Principles	Application in the Food Industry
Value creation and protection	This component emphasizes the importance of creating and protecting value within the food industry. This involves not only generating profit but also ensuring that ethical considerations are prioritized to protect the well-being of consumers, workers, and the environment.
Integrated	The food industry must integrate ethical considerations into all aspects of its operations, including production, distribution, marketing, and waste management. This integration ensures that ethical values are embedded throughout the entire supply chain.
Structured	Ethical decision-making processes should be structured and systematic, guided by clear principles and guidelines. This ensures consistency and transparency in how ethical dilemmas are addressed within the food industry.
Customized	Recognizing that different contexts may require tailored ethical approaches, the food industry should customize its ethical practices to suit specific situations, regions, or cultural norms. This flexibility allows for more effective and culturally sensitive ethical decision-making.
Inclusive	Ethical practices in the food industry should be inclusive, considering the perspectives and needs of all stakeholders, including consumers, producers, workers, communities, and regulatory bodies. Inclusivity fosters collaboration and ensures that diverse voices are heard in ethical decision-making processes.
Dynamic and responsive	Ethical considerations in the food industry should be dynamic and responsive to changing circumstances, emerging issues, and stakeholder feedback. This adaptability enables the industry to address new challenges and seize opportunities for improvement.
Best available information	Ethical decision-making in the food industry should be informed by the best available information, including scientific research, industry standards, consumer preferences, and expert advice. This ensures that decisions are based on evidence and expertise rather than speculation or bias.
Human and cultural factors	Ethical practices in the food industry should consider the human and cultural factors that influence food consumption, production, and distribution. This includes considerations of food traditions, dietary preferences, labor rights, and social norms.
Continual improvement	The food industry should strive for continual improvement in its ethical practices, seeking to raise standards, address shortcomings, and innovate new solutions. This commitment to ongoing improvement ensures that ethical considerations remain at the forefront of industry efforts.

**Table 3:** ISO standards for ethical practices in the food industry.

ISO Standard	Title
ISO 26000: 2010 https://www.iso.org/iso-26000-social-responsibility.html, accessed 12 April 2024.	Guidance on social responsibility
ISO 22000:2018 https://www.iso.org/standard/65464.html accessed 12 April 2024	Food safety management systems
ISO 14001:2015 https://www.iso.org/standard/60857.html accessed 12 April 2024	Environmental management systems
ISO 9001: 2015 [164]	Quality management systems
ISO 31000:2018 [165]	Risk management
ISO 45001:2018 https://www.iso.org/standard/63787.html accessed 12 April 2024	Occupational health and safety management systems
ISO 20400:2017 https://www.iso.org/obp/ui/en/#iso:std:iso:20400:ed-1:v1:en accessed 12 April 2024	Sustainable procurement
ISO 50001:2018 https://www.iso.org/standard/69426.html accessed 12 April 2024	Energy management
ISO 22301:2019https://www.iso.org/standard/75106.html accessed 12 April 2024	Security and resilience
ISO 27001:2018 https://www.iso.org/standard/73906.html accessed 12 April 2024	Information security management system (ISMS)
ISO 37001:2016 https://www.iso.org/standard/65034.html accessed 12 April 2024	Anti-bribery management systems

**Table 4:** Possible food frauds and consumer countermeasures. Adapted from [204,205].

Food Fraud Type	Description	Consumer Countermeasures
Mislabeling	Deliberate substitution, addition, tampering, or false/misleading statements for gain	Demand transparency in labeling and certification processes, verify product authenticity, and report suspicions
Adulteration	Addition of unauthorized substances like formalin, urea, starch, etc., for economic gain	Support stringent quality control measures, seek products with reputable certifications, and report suspicions
Lack of trust in the supply chain	Distrust among supply chain actors leads to increased vulnerability to fraud	Choose products from transparent and accountable supply chains, support ethical brands, and demand traceability
Opportunistic behavior	Supply chain partners exploiting situations for personal gain	Enhance fair business practices and endorse initiatives fostering integrity and accountability
Inadequate supply chain governance	Poor oversight and control mechanisms within the supply chain, enabling fraudulent activities	Advocate for regulatory reforms and support initiatives enhancing governance and accountability
Complexity of the supply chain	Complexity of supply chain operations contributing to increased risk of fraud	Support simplified and transparent supply chain structures and favor local and short supply chains
Government regulation	Insufficient regulatory frameworks and enforcement contribute to fraud vulnerabilities	Implement stricter regulations and enforcement and support initiatives promoting regulatory compliance
Social governance	Social factors influencing fraud susceptibility within the supply chain	Promote consumer awareness and education and support initiatives fostering social responsibility and transparency
Detection techniques	Inadequate fraud detection methods and technologies allow fraud to go undetected	Invest in advanced detection technologies, support initiatives improving fraud detection, and share information

**Table 5:** Significance of oral health in the food chain adapted from [358].

Aspect of Oral Health	Significance
Digestion	Begins in the mouth through chewing (mastication), which breaks down food into smaller particles, facilitating digestion and nutrient absorption. Saliva, produced by salivary glands, contains enzymes that initiate carbohydrate breakdown.
Respiratory system	The mouth and nasal cavities are interconnected with the respiratory system, facilitating proper breathing and oxygen intake.
Pathogen defense	The mouth acts as a primary entry point for pathogens, bacteria, and foreign substances, making oral hygiene crucial for preventing infections and maintaining overall health.
Oral health issues and systemic health	Poor oral hygiene can lead to various oral health issues such as dental caries, periodontal diseases, and oral infections, which can have systemic implications for overall health.
Interdisciplinary collaboration	Emphasizes the need for collaboration between dental and medical professionals to effectively address the bidirectional relationship between oral health and systemic health.
Oral microbiome	The oral microbiome plays a critical role in both oral and systemic diseases, influencing various conditions such as cardiovascular disease, diabetes, and even neurodegenerative disorders.
Saliva as a diagnostic medium	Saliva serves as a valuable tool for understanding oral and systemic health, with ongoing research focusing on its potential for point-of-care testing and disease monitoring.

## Data Availability

No new data were created or analyzed in this study. Data sharing does not apply to this article.
